# Overexpressing Exogenous 5-Enolpyruvylshikimate-3-Phosphate Synthase (EPSPS) Genes Increases Fecundity and Auxin Content of Transgenic Arabidopsis Plants

**DOI:** 10.3389/fpls.2018.00233

**Published:** 2018-02-27

**Authors:** Jia Fang, Peng Nan, Zongying Gu, Xiaochun Ge, Yu-Qi Feng, Bao-Rong Lu

**Affiliations:** ^1^Ministry of Education Key Laboratory for Biodiversity and Ecological Engineering, Department of Ecology and Evolutionary Biology, Fudan University, Shanghai, China; ^2^State Key Laboratory of Genetic Engineering, Department of Biochemistry and Molecular Biology, School of Life Sciences, Institute of Plant Biology, Fudan University, Shanghai, China; ^3^Key Laboratory of Analytical Chemistry for Biology and Medicine, Ministry of Education, Department of Chemistry, Wuhan University, Wuhan, China

**Keywords:** abiotic stress, *Arabidopsis thaliana*, fitness, glyphosate-tolerance, growth hormone, indole-3-acetic acid, seed germination, transgenic plant

## Abstract

Transgenic glyphosate-tolerant plants overproducing EPSPS (5-enolpyruvylshikimate-3-phosphate synthase) may exhibit enhanced fitness in glyphosate-free environments. If so, introgression of transgenes overexpressing *EPSPS* into wild relative species may lead to increased competitiveness of crop-wild hybrids, resulting in unpredicted environmental impact. Assessing fitness effects of transgenes overexpressing *EPSPS* in a model plant species can help address this question, while elucidating how overproducing EPSPS affects the fitness-related traits of plants. We produced segregating T_2_ and T_3_ *Arabidopsis thaliana* lineages with or without a transgene overexpressing *EPSPS* isolated from rice or *Agrobacterium* (*CP4*). For each of the three transgenes, we compared glyphosate tolerance, some fitness-related traits, and auxin (indole-3-acetic acid) content in transgene-present, transgene-absent, empty vector (EV), and parental lineages in a common-garden experiment. We detected substantially increased glyphosate tolerance in T_2_ plants of transgene-present lineages that overproduced EPSPS. We also documented significant increases in fecundity, which was associated with increased auxin content in T_3_ transgene-present lineages containing rice *EPSPS* genes, compared with their segregating transgene-absent lineages, EV, and parental controls. Our results from Arabidopsis with nine transgenic events provide a strong support to the hypothesis that transgenic plants overproducing EPSPS can benefit from a fecundity advantage in glyphosate-free environments. Stimulated biosynthesis of auxin, an important plant growth hormone, by overproducing EPSPS may play a role in enhanced fecundity of the transgenic Arabidopsis plants. The obtained knowledge is useful for assessing environmental impact caused by introgression of transgenes overproducing EPSPS from any GE crop into populations of its wild relatives.

## Introduction

Genetically engineered (GE) herbicide-tolerant crops are cultivated extensively over the world owing to their substantial agronomic, environmental, economic, health and social benefits (James, 2016). Herbicide-tolerant GE crops occupy ~76% of the total GE crop cultivation area, including herbicide-tolerant and stacked herbicide-tolerant/insect-resistant GE crops (James, 2016). Of these, glyphosate-tolerance represents the world's most widespread GE crop trait (Duke and Powles, 2008; Vats, 2015; James, 2016). The commercial cultivation of glyphosate-tolerant GE crops has greatly promoted the glyphosate application in agricultural ecosystems, consequently arousing global concerns over its potential environmental impact. Many weed species have evolved glyphosate tolerance under selective pressure after long-term glyphosate applications (Duke and Powles, 2008; Délye et al., 2013). Glyphosate-selective-pressure induced target-site mutations (Gaines et al., 2010, 2011; Chen et al., 2015) and amplification of the *EPSPS* genes (Nandula et al., 2013; Sammons and Gaines, 2014) have been found in those resistant weeds resulting new environmental problems as farmers shift to less environmentally friendly herbicides. In addition, transgene flow from glyphosate-tolerant GE plants to populations of wild or weedy relatives has been found, becoming an environmental biosafety concern (Reichman et al., 2006; Warwick et al., 2008; Wegier et al., 2011; Zapiola and Mallory-Smith, 2017). Yet, some researchers posit little or no environmental impact from introgression of glyphosate-tolerance transgenes into wild relative populations because they believe that such transgenes offer no fitness advantage in natural ecosystems in the absence of glyphosate (Cerdeira and Duke, 2006; Vila-Aiub et al., 2014).

The findings of significantly enhanced fecundity of crop-weed (Wang et al., 2014) and crop-wild (Yang et al., 2017b) rice hybrid progeny containing the glyphosate-tolerance transgene in a glyphosate-absent habitat suggests that introgression of such a glyphosate-tolerance transgene might result in transgene persistence and spread in populations of wild relatives, possibly causing environmental consequences (Qiu, 2013; Ryffel, 2014; Vila-Aiub et al., 2015; Martin et al., 2017). Glyphosate can competitively inhibit EPSPS (5-enolpyruvylshikimate-3-phosphate synthase, EC 2.5.1.19), resulting in weakness or even death of plants at the proper dosages (Roberts et al., 1998; Mueller et al., 2003; Duke and Powles, 2008). EPSPS is a key enzyme in the shikimate pathway, which is extremely important because ~35% or more plant biomass in the form of dry matter is represented by aromatic molecules derived directly from this pathway (Franz et al., 1997). In addition, EPSPS is essential for the production of aromatic amino acids (e.g., tryptophan, phenylalanine, and tyrosine) and other secondary metabolites (Weaver and Herrmann, 1997), suggesting that EPSPS is crucial for the survival, growth, and development of plants.

Overproduction of EPSPS, attempting to provide sufficient surplus of EPSPS binding to glyphosate to reduce its fatal toxicity, is one of the strategies to increase plants' tolerance to glyphosate. This strategy includes developing GE plants with multiple copies of the *EPSPS* gene (Rogers et al., 1983; Goldsbrough et al., 1990; Shyr et al., 1992) and increasing basal levels of the EPSPS enzyme by fusing an *EPSPS* gene with a strong promotor (Klee et al., 1987; Su et al., 2008). Notably, certain GE glyphosate-tolerant crops overexpressing *EPSPS* not only have increased glyphosate tolerance (Klee et al., 1987; Su et al., 2008; Vats, 2015; Yang et al., 2017a), but unexpectedly also showed increased yield (Zhou et al., 2003; Owen et al., 2010) and other fitness traits. The increased yield can also be manifest as increased fecundity in GE hybrid progeny with weedy (*O. sativa* f. *spontanea*, Wang et al., 2014) and wild (*O. rufipogon*, Yang et al., 2017b) rice overexpressing *EPSPS*, even under glyphosate-free conditions. Wang et al. (2014) also reported significantly increased Trp concentrations in crop-weed F_2_ transgene-present hybrid lineages of the GE rice (*Oryza sativa*) line overexpressing *EPSPS* and four weed rice populations in the glyphosate-free environment. It has been proven that Trp is associated with the biosynthesis of plant growth hormone auxin (Zhao, 2012). In addition, Yang et al. (2017b) reported considerably altered phenology of F_2_ *EPSPS* transgene-present hybrid lineages with two wild rice populations in the glyphosate-free environment. Altogether, these findings suggest that transgenes overproducing EPSPS will change fitness of crop-weed/wild hybrids.

**Figure 1:**
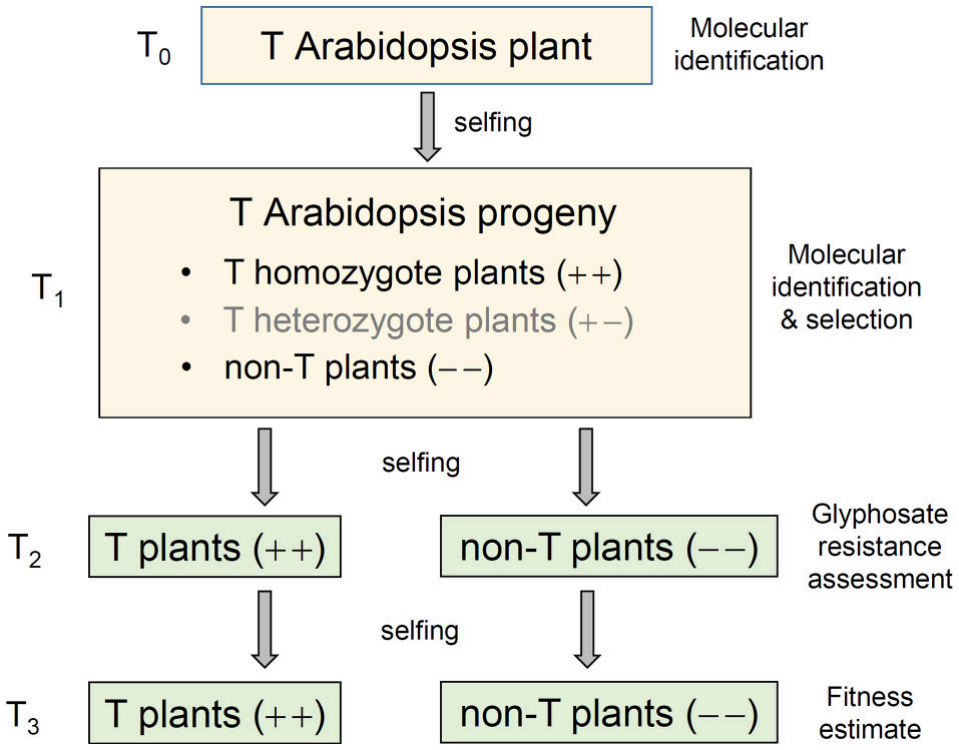
A schematic pedigree to illustrate the production of transgenic (T) Arabidopsis progeny containing genes overexpressing the 5-enolpyruvoylshikimate-3-phosphate synthase (EPSPS). A T_0_ transgenic Arabidopsis plant was self-pollinated to produce the T_1_ progeny that contained transgene-homozygote (+ +), transgene-heterozygote (+ –), and transgene-absent (– –) plants. The isogenic transgene-homozygote (+ +) and transgene-absent (– –) lineages generated from T_1_ plants through self-pollination (selfing) and molecular identification were retained for the experiments of glyphosate tolerance (T_2_), and *EPSPS* transgene expression, biomass and auxin (IAA) content, and fitness assessment (T_3_).

Gressel et al. (2014) and Grunewald and Bury (2014) questioned whether the enhanced fecundity and metabolic traits in the glyphosate-tolerant crop-weed hybrids overexpressing *EPSPS* was due to the position effect of the gene insertion or the possible linkage with neighboring sequences because only a single transgenic event was involved in the study of Wang et al. (2014). It is therefore critical to confirm the observed increases in fecundity and metabolic traits are caused by the *EPSPS* transgene *per-se*, rather than the other actions of transgenes. In addition, it is necessary to address whether the observed changes in fecundity by overexpression of the *EPSPS* transgene is a general phenomenon? In other words, can the phenomenon observed in rice (*Oryza*) be also found in very distant plant species such as Arabidopsis? To answer the above question, we produced multiple independent events of transgenic *Arabidopsis thaliana* plants overexpressing exogenous *EPSPS* genes from different sources (rice and *Agrobacterium*), driven by a cauliflower mosaic virus 35S promotor (pCaMV35S). We also produced transgenic plants only containing the selective marker gene (*nptII*) driven by pCaMV35S as the empty vector (EV) control. The T_2_ and T_3_ segregating transgene-present and transgene-absent lineages, EV and parent controls were compared to estimate differences in glyphosate tolerance and fitness-related traits (Figure 1) to test whether the observed changes in rice would also occur in a phylogenetically distant plant species.

The objectives of this study are to address the following questions: (1) Does overproduction of EPSPS increase glyphosate tolerance of the Arabidopsis plants in transgenic lineages? (2) Does overexpression of exogenous *EPSPS* genes enhance fitness of transgenic Arabidopsis plants? (3) If so, is enhanced fitness of the transgenic plants caused by the position effect of an inserted gene? (4) Do transgene-present lineages that overproduce EPSPS synthesize more auxin than transgene-absent lineages? Answers to these questions will increase our understanding of the general effects of overexpressing *EPSPS* genes on the phenotypes of plant species.

## Materials and methods

### *Arabidopsis thaliana* transgenic lineages

An *A. thaliana* strain Columbia (Col 0, coded as P) was used as the parent to produce the comparative transgene-present and transgene-absent lineages in this study. Three *EPSPS* genes were used to develop transgenic constructs: two isolated from cultivated rice (Zhou et al., 2006; Su et al., 2008) and one (*CP4*, coded as C) from an *Agrobacterium* sp. strain (Padgette et al., 1996). The two genes from rice included one normal *EPSPS* (coded as E, Zhou et al., 2006) and another mutant *EPSPS* that was a C^317^-T mutation (coded as Em, Su et al., 2008). This Em gene was obtained by an error-prone PCR (polymerase chain reaction) technique and an Em transgenic rice line conferred high level of tolerance to glyphosate (Su et al., 2008; Lu et al., 2014a,b). The vector pCHF3 was used for overexpression transgenic constructs that contained one of the target *EPSPS* genes and a selective marker gene (*nptII*), both driven by pCaMV35S, respectively (Figure S1, upper panel). The *nptII* (neomycin phosphotransferase) marker gene conferred tolerance to kanamycin. An empty vector only including the selective marker gene (*nptII*) driven by pCaMV35S was also developed (Figure S1, lower panel) as a control.

The transgenic constructs were introduced into *Agrobacterium tumefaciens* first and then transformed into Arabidopsis plants by the floral-dip method (Clough and Bent, 1998) for genetic transformation. All seeds from the transformation-treated Arabidopsis plants were germinated on the 1/2 MS culture media, including 30 g sucrose, 2.2 g M519 (Murashige & Skoog Basal Medium with Vitamins), 50 ng kanamycin, and 8 g agar per liter (pH 5.7). All survived plants (T_0_) from the 1/2 MS culture media were subjected to molecular identification for the target and marker transgenes ($Appendix S2) to confirm their transgenic status. The primer sequences for the *EPSPS* and mutant *EPSPS* transgenes were 5′-acgaatgagggagagaccga-3′ and 5′-accatcagcgaagagtgcaa-3′; whereas those for the *CP4* transgene were 5′-gcgtcgccgatgaaggtgctgt-3′ and 5′-cggtccttcatgttcggcggtctc-3′. Primer sequences for the *nptII* marker gene were 5′-taaagcacgaggaagcggtc-3′ and 5′-gatggattgcacgcaggttc-3′. All the primers were synthesized by the Shanghai Sangon Biotech Co., Ltd (Shanghai, China). To retain transgenic plants that are presumed to have a single copy of the transgenes (E, Em, or C), we selected T_1_ progeny generated from self-pollination of a T_0_ transgenic plant with a 3: 1 segregation ratio for kanamycin tolerant: sensitive, and confirmed by molecular identification (Figure S2) for further experiments.

To determine the transgene insertion or position effect causing the fitness change, we retained three transgenic events from each of the three transgenic constructs (E, Em, or C) with the above-average level of *EPSPS* expression for each transgenic construct in the T_2_ and T_3_ generations for further analyses (Appendix S3). The reason for measuring transgenic events in T_2_ and T_3_ was to examine the stability of *EPSPS* expression between generations. Transgenic events with the highest level of *EPSPS* expression in the T_2_ and T_3_ generations (Table S1) were excluded from the experiment to avoid the extreme cases. We only retained the isogenic lineages with transgenic homozygote (+ +) and transgene-absent plants (– –) from T_1_ segregating populations after molecular identification and selection for the transgenes in the T_2_ generation (Figure 1, Appendix S2). A total of 20 Arabidopsis lineages, including three transgene-present lineages (E+, Em+, and C+) for each of the three events (9), three segregated transgene-absent lineages (E–, Em–, and C–) for each of the three events each representing the three transgenes (9), one empty vector lineage (EV), and one parental strain (P), were used for further experiments.

### Glyphosate tolerance

Twenty Arabidopsis lineages in the T_2_ generation (Figure 1) were used to test glyphosate (Roundup®, glyphosate isopropyl amine salt aqueous solution: 41%) tolerance in a dose-response experiment. For each lineage (event, EV, or parent), three replicates (*n* = 3) each included three plants grown in a growth chamber (22°C), were included. A total of 180 plants were included in the test for glyphosate tolerance for the 20 lineages at each glyphosate concentration. Forty days after seeds were sown, all plants were sprayed with glyphosate aquatic solution at nine different concentrations: 0, 0.1, 0.2, 0.4, 0.8, 1.0, 1.2, 2.0, 5.0 m*M*, in which 0.4 m*M* equivalent to the concentration of 840 g/ha is the commonly used dosage for glyphosate application in fields for the agricultural weed control (Norsworthy et al., 2008). Therefore, a total of 1,620 plants was used in the glyphosate tolerance experiment. Survival ratios were determined as the number of surviving Arabidopsis plants as percent of the total number of plants used for analyses 15 days after glyphosate application. Plants that were partially green but with green meristems and central rosettes were scored as “surviving;” plants that had white meristems and central rosettes were scored as “dead” (Figure S3).

### *EPSPS* transgene expression and EPSPS protein content

Twenty Arabidopsis lineages in the T_3_ generation (Figure 1) were used to determine *EPSPS* transgene expression and EPSPS protein content. Each Arabidopsis plant in the T_3_ generation was equally divided as two parts for measuring *EPSPS* transgene expression and the EPSPS protein content, respectively. For each of the 20 lineages (6 E+ and E–, 6 Em+ and Em–, 6 C+ and C–, 1 EV and 1 P), a pooled sample (each including 8 plants) with three replicates (*n* = 3) were used for these measurements 30 days after seeds were sown. Therefore, a total of 480 plants was used in this experiment.

Real-time PCR was applied to determine the expression of *EPSPS* transgene relative to an *ubiquitin* reference gene (*UBQ*) from Arabidopsis. Total RNA was isolated from the 30-day-old plants using the RNAsimple Total RNA kit (TianGen, Beijing, China). DNA removal and RNA reverse-transcription were conducted using the PrimeScript^®;^ RT reagent Kit with gDNA Eraser (TaKaRa, Dalian, China). The primers for real-time PCR were designed using the software Primer PREMIER ver. 5.0. The primer sequences for the *EPSPS* and mutant *EPSPS* transgenes were 5′-aaggatgcgaaagagg-3′ and 5′-caacccgacaaccaa-3′; whereas those for the *CP4* transgene were 5′-tggattgcgatgaggg-3′ and 5′-tgatcgagatgggtggc-3′. The primer sequences for the reference gene (*UBQ*) were 5′-aatgtgaaggcgaagatccaagac-3′ and 5′-agacggaggacgagatgaagc-3′. The expression of the *EPSPS* genes in non-transgenic, EV, and parental lineages was measured using the Arabidopsis endogenous *EPSPS* gene. The primer sequences for the Arabidopsis endogenous *EPSPS* gene were 5′-aacgcaagttatgtcc-3′ and 5′-gcagttagtgccaag-3′. All the primers were synthesized by the Shanghai Sangon Biotech Co., Ltd. (Shanghai, China). The real-time PCR reaction mixture kit (TaKaRa, Dalian, China) included 1 μl of template cDNA, 0.4 μl each of the forward and reverse 10 μM primers, and 1 × SYBR^®;^Premix Ex Taq^TM^ in a final volume of 20 μl. PCR reaction was conducted at 94°C 30 s; 94°C 15 s, 60°C 15 s, and 40 s at 72°C for 40 cycles.

The sandwich technique of ELISA (enzyme linked immunosorbent assay) was performed to determine the EPSPS protein content. The total proteins were extracted based on the method of phosphate-buffered saline (PBS, 28.7 g Na_2_HPO_4_-12H_2_O and 2.96 g NaH_2_PO_4_-2H_2_O, pH 7.4) with 10% (v/v) methanol in 0.1 M PBS to suspend the samples after ground into powder in liquid nitrogen. The samples were then incubated in ice bath for 40 min, followed by centrifuged at 8,000 × g, at 4°C for half an hour. The supernatant was collected for EPSPS protein content determination with the Quantiplate kit (Envirologix, Portland, OR, USA) for detecting EPSPS protein in plants and bacteria following the manufacturer's instructions. The Microplate Reader (Bio-Rad Laboratories, Inc., Hercules, CA, USA) was used to detect the optical density (OD) at the wavelength of 450 nm.

### Fitness-related traits

Twenty Arabidopsis lineages in the T_3_ generation were used to estimate fitness (Figure 1). Nine fitness-related traits were used for the measurement at different times: seed germination under normal and stressed conditions, leaf area, plant height and branching, the number of siliques per plant, number of seeds per silique and per plant (Table S2). To estimate seed germination ratios for each lineage, six replicate samples (*n* = 6) each with 50 seeds were germinated on 1/2 MS culture media under the normal, heat, and drought stress conditions, respectively (Table S2). To estimate the other fitness-related traits (Appendix S5), six replicates (*n* = 6) each with four plants were grown in each of the 20 lineages in a growth chamber (22°C). The layout of the total 120 replicates (pots) followed a completely randomized design.

### Biomass and auxin content

Six Arabidopsis lineages with or without the *EPSPS* transgenes (E, Em, and C) in the T_3_ generation (Figure 1; Table S1) were used to measure biomass and auxin content. The auxin content was determined as the average total weight (ng) of auxin in plants at the same growth stage. One transgenic event (E2, Em3, or C2) each representing one of the three transgenic constructs (see Table S1) was randomly selected to measure plant biomasses and the auxin content, using fresh samples of the entire 30-day-old plants. From each event, five independent replicates (*n* = 5), each represented by five different plants of transgene-present or transgene-absent lineages, were measured for the biomass and auxin content (a pooled sample from five plants per replicate, Table S2), following the methods of Chen et al. (2012). A total of 150 plants were used for determining biomasses and the auxin content. The empty vector and parental lineages were not included for analyses.

### Statistical analyses

One way ANOVA was conducted to examine the effect of the presence or absence of each *EPSPS* transgene, involving transgene-present (E+, Em+, or C+), transgene-absent (E–, Em–, or C–), EV, and parental lineages. One way ANOVA was also conducted to determine the effect of different events from each transgenic construct (E, Em, or C) on fitness-related traits. Duncan's multiple range test was conducted to determine significant differences in gene expression, protein content, and the fitness-related traits that showed the significant transgenic effect among transgene-present, transgene-absent, EV, and parental lineages based on one way ANOVA. The independent *t*-tests with Bonferroni corrections were conducted to test for significant differences in all measured fitness-related traits and auxin content between transgene-present and transgene-absent lineages, after the measured values were subject to the Levene's test for equality of variances. All statistical analyses were performed using the software SPSS ver. 19.0 (IBM Inc., New York, USA).

## Results

### Glyphosate tolerance

Substantially increased tolerance to glyphosate was detected in Arabidopsis plants of transgene-present lineages of all events representing the three transgenic constructs, compared to those of their transgene-absent, EV, and parental lineages in the T_2_ generation (Table 1; Figure S3a,b). The three types of transgenic Arabidopsis plants (E+, Em+, and C+) survived at different concentrations (0.1–1.2 m*M*) of glyphosate, although with some degrees of variation (~10–35%) among transgenic constructs at the same concentration. More than 50% transgenic plants of the three constructs survived when the concentration of glyphosate increased to 1.2 m*M* (Table 1). However, none of the transgene-absent Arabidopsis plants (E–, Em–, and C–) survived at the concentration of 0.4 m*M* glyphosate (Table 1), which was equivalent to the commonly applied glyphosate dosage (840 g/ha) in the field. Notably, ~7% plants in the transgenic *CP4* lineages (C+) survived at the concentration of 2.0 m*M* glyphosate.

**Table 1:** Average survival ratios (%) of *Arabidopsis thaliana* plants in T_2_ transgene-present (E+, Em+, or C+) lineages overexpressing *EPSPS* and their segregating transgene-absent (E–, Em–, or C–), empty vector (EV), and parental (P) lineages under different glyphosate concentrations.

**Event/ lineage**	**Survival ratio (%) of Arabidopsis plants under different glyphosate concentration (m*****M*****)**								
	**0**	**0.1**	**0.2**	**0.4^a^**	**0.8**	**1.0**	**1.2**	**2.0**	**5.0**
E+/2	100	100	100	**100**	100	88.9 (11.1)	77.8 (11.1)	0	0
E–/2	100	100	55.6 (11.1)	**0**	0	0	0	0	0
E+/3	100	100	100	**100**	88.9 (11.1)	66.7 (19.2)	55.6 (11.1)	0	0
E–/3	100	100	66.7 (19.2)	**0**	0	0	0	0	0
E+/4	100	100	100	**100**	88.9 (11.1)	77.8 (11.1)	55.6 (11.1)	0	0
E–/4	100	100	77.8 (11.1)	**0**	0	0	0	0	0
Em+/2	100	100	100	**100**	100	88.9 (11.1)	77.8 (11.11)	0	0
Em–/2	100	100	66.7 (0)	**0**	0	0	0	0	0
Em+/3	100	100	100	**100**	100	77.8 (11.1)	66.7 (19.25)	0	0
Em–/3	100	100	66.7 (19.2)	**0**	0	0	0	0	0
Em+/4	100	100	100	**100**	88.9 (11.1)	77.8 (11.1)	55.6 (11.1)	0	0
Em–/4	100	100	33.3 (19.2)	**0**	0	0	0	0	0
C+/2	100	100	100	**100**	100	100	88.9 (11.1)	22.2 (11.1)	0
C–/2	100	100	55.6 (11.1)	**0**	0	0	0	0	0
C+/3	100	100	100	**100**	88.9 (11.1)	66.7 (19.2)	66.7 (19.2)	0	0
C–/3	100	100	77.8 (11.1)	**0**	0	0	0	0	0
C+/4	100	100	100	**100**	100	88.9 (11.1)	66.7 (19.2)	0	0
C–/4	100	100	55.6 (11.1)	**0**	0	0	0	0	0
EV	100	100	59.3 (9.1)	**0**	0	0	0	0	0
P	100	100	62.9 (10.6)	**0**	0	0	0	0	0

*Numbers in parenthesis indicate standard errors (n = 3 replicates with three plants per replicate)*.

a *0.4 mM (bold fonts) is equivalent to the concentration of 840 g ae/ha, which is the commonly used dosage for glyphosate application in fields for agricultural weed control (Norsworthy et al., 2008)*.

### Expression of *epsps* transgenes and EPSPS protein content

Significantly increased expression of the three *EPSPS* transgenic constructs (E, Em, and C) and content of the EPSPS proteins were detected in Arabidopsis plants of transgene-present lineages, compared to those of transgene-absent, EV, and the parental lineages (Table 2). The average values of relative expression of the three transgenes: *EPSPS*, mutant *EPSPS*, and *CP4*, measured by real-time PCR were significantly greater (*P* < 0.01) in the T_3_ transgene-present lineages (E+, Em+, and C+) than those in their corresponding transgene-absent (E–, Em–, and C–), the EV, and parental lineages based on the Duncan's multiple range test (Table 2). In addition, the average values of EPSPS protein content measured by ELISA were also significantly greater (*P* < 0.05) in the T_3_ transgene-present lineages (E+, Em+, and C+) than those in their corresponding transgene-absent (E–, Em–, and C–), the EV and parental lineages based on the Duncan's multiple range test (Table 2).

**Table 2:** Means of relative transgene expression and EPSPS protein content in T_3_ *Arabidopsis thaliana* plants of transgene-present (E+, Em+, and C+), transgene-absent (E–, Em–, and C–), empty vector (EV), and the parental lineages, measured by real-time PCR (polymerase chain reaction) and ELISA (enzyme linked immunosorbent assay), respectively.

**Lineage**	**Relative gene expression in different events^1^**			**Protein content (ng/ml) in different events^2^**		
**Event ID:**	**2**	**3**	**4**	**2**	**3**	**4**
***EPSPS***						
E+	**6.609 (0.943)**^A^	4.354 (0.320)^A^	3.022 (0.353)^A^	199.48 (0.66)^a^	198.76 (0.14)^a^	202.64 (0.80)^a^
E–	**0.012 (0.002)**^B^	0.007 (0.001)^B^	0.006 (0.001)^B^	146.74 (1.33)^c^	149.68 (0.52)^c^	156.06 (1.04)^c^
EV	0.010 (0.004)^B^	0.010 (0.004)^B^	0.010 (0.004)^B^	178.26 (0.43)^b^	178.26 (0.43)^b^	178.26 (0.43)^b^
P	0.008 (0.002)^B^	0.008 (0.002)^B^	0.008 (0.002)^B^	177.51 (3.10)^b^	177.51 (3.10)^b^	177.51 (3.10)^b^
**MUTANT** ***EPSPS***						
Em+	6.008 (0.824)^A^	**4.725 (0.734)**^A^	1.000 (0.479)^A^	219.91 (0.38)^a^	228.26 (0.14)^a^	219.91 (0.80)^a^
Em–	0.054 (0.015)^B^	**0.035 (0.002)**^B^	0.018 (0.001)^B^	182.21 (0.25)^b^	183.65 (1.88)^b^	176.74 (0.88)^b^
EV	0.010 (0.004)^B^	0.010 (0.004)^B^	0.010 (0.004)^B^	178.26 (0.43)^b^	178.26 (0.43)^b^	178.26 (0.43)^b^
P	0.008 (0.002)^B^	0.008 (0.002)^B^	0.008 (0.002)^B^	177.51 (3.10)^b^	177.51 (3.10)^b^	177.51 (3.10)^b^
***CP4***						
C+	**6.585 (0.476)**^A^	4.228 (0.226)^A^	1.331 (0.321)^A^	211.23 (0.99)^a^	205.66 (1.01)^a^	209.41 (0.25)^a^
C–	**0.026 (0.005)**^B^	0.018 (0.001)^B^	0.014 (0.003)^B^	178.26 (0.50)^b^	182.14 (0.90)^b^	176.54 (0.66)^b^
EV	0.010 (0.004)^B^	0.010 (0.004)^B^	0.010 (0.004)^B^	178.26 (0.43)^b^	178.26 (0.43)^b^	178.26 (0.43)^b^
P	0.008 (0.002)^B^	0.008 (0.002)^B^	0.008 (0.002)^B^	177.51 (3.10)^b^	177.51 (3.10)^b^	177.51 (3.10)^b^

*Numbers in parenthesis indicate standard errors (n = 3 replicates, each including eight different plants as a pooled sample). Events with bold letters indicate those used for biomass and auxin examination*.

1 *Different capital letters indicate significances at the 0.01 level*.

2 *Different small letters indicate significances at the 0.05 level*.

### Fecundity and other fitness-related traits

We first tested differences in all included fitness-related traits among the three transgenic events based on each transgene-present or transgene-absent lineage using one-way ANOVA. Because none of these traits showed significant differences among the transgenic events (*P* < 0.05, Table S3), we grouped data from three events each with six replicates (*n* = 18) for each transgenic construct in subsequent analyses (Table S4). Thus, for seed germination, we analyzed data from 1,800 seeds per transgene for each treatment, while for other fitness-related traits, we analyzed data from 144 plants per transgene. One-way ANOVAs indicated significant effects of the presence versus absence of the three transgenes (E, Em, or C) on some fitness-related traits in the T_3_ generation (Table 3), including relative leaf area, plant height, and the numbers of siliques and seeds per plant (Table 3).

**Table 3:** One-way ANOVA to test the effects of each of the three *epsps* transgenes on fitness-related traits in T_3_ *Arabidopsis thaliana* plants, including the transgene-present (3), transgene-absent (3), empty vector (1), and parental (1) lineages.

**Trait**	***EPSPS*** **(E)**			***Mutant EPSPS*** **(Em)**			***CP4*** **(C)**		
	**df**	***F***	***P***	**df**	***F***	***P***	**df**	***F***	***P***
Seed germination under normal condition	3	1.84	0.218	3	0.43	0.737	3	1.61	0.262
Seed germination under heat stress	3	21.83	<**0.001**	3	12.86	<**0.001**	3	0.67	0.576
Seed germination under drought stress	3	15.66	**0.001**	3	14.08	**0.001**	3	0.87	0.495
Relative leaf area	3	8.40	<**0.001**	3	5.32	**0.003**	3	3.85	**0.016**
Plant height at maturity	3	3.92	**0.014**	3	1.33	0.278	3	3.14	**0.035**
Number of branches per plant	3	0.98	0.412	3	1.32	0.279	3	0.96	0.420
Number of siliques per plant	3	5.95	**0.002**	3	6.36	**0.001**	3	5.22	**0.004**
Number of seeds per silique	3	2.01	0.126	3	2.96	**0.042**	3	1.08	0.366
Number of seeds per plant	3	7.23	<**0.001**	3	9.46	<**0.001**	3	5.19	**0.004**

*For seed germination, each lineage included six replicates (n = 6) each containing 50 seeds. For other traits, each lineage included six replicates (n = 6) each containing four plants. Bold values indicate the significant traits*.

**Figure 2:**
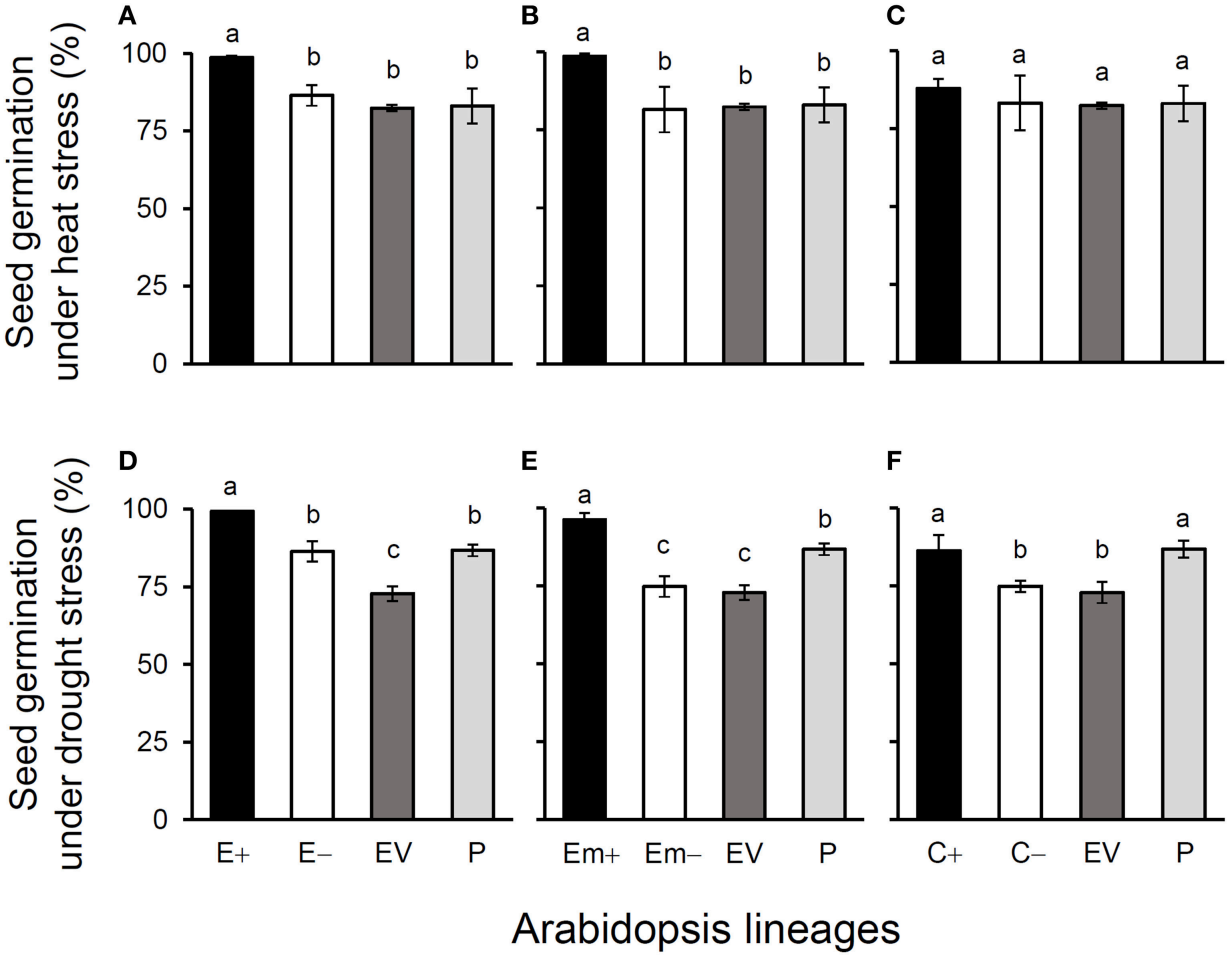
Average % seed germination of three Arabidopsis transgenic events under heat **(A–C)** and drought **(D–F)** stresses in T_3_ transgene-present, transgene-absent, empty vector (EV), and parent (P) lineages in the glyphosate-free environment. Different letters above the columns indicate significances at *P* < 0.05 based on Duncan's multiple range test (*n* = 18). E+: *EPSPS* transgene-present lineages, E–: *EPSPS* transgene-absent lineages, Em+: mutant *EPSPS* transgene-present lineages, Em–: mutant *EPSPS* transgene-absent lineages; C+: *CP4* transgene-present lineages, C–: *CP4* transgene-absent lineages. Bars represent standard errors.

**Figure 3:**
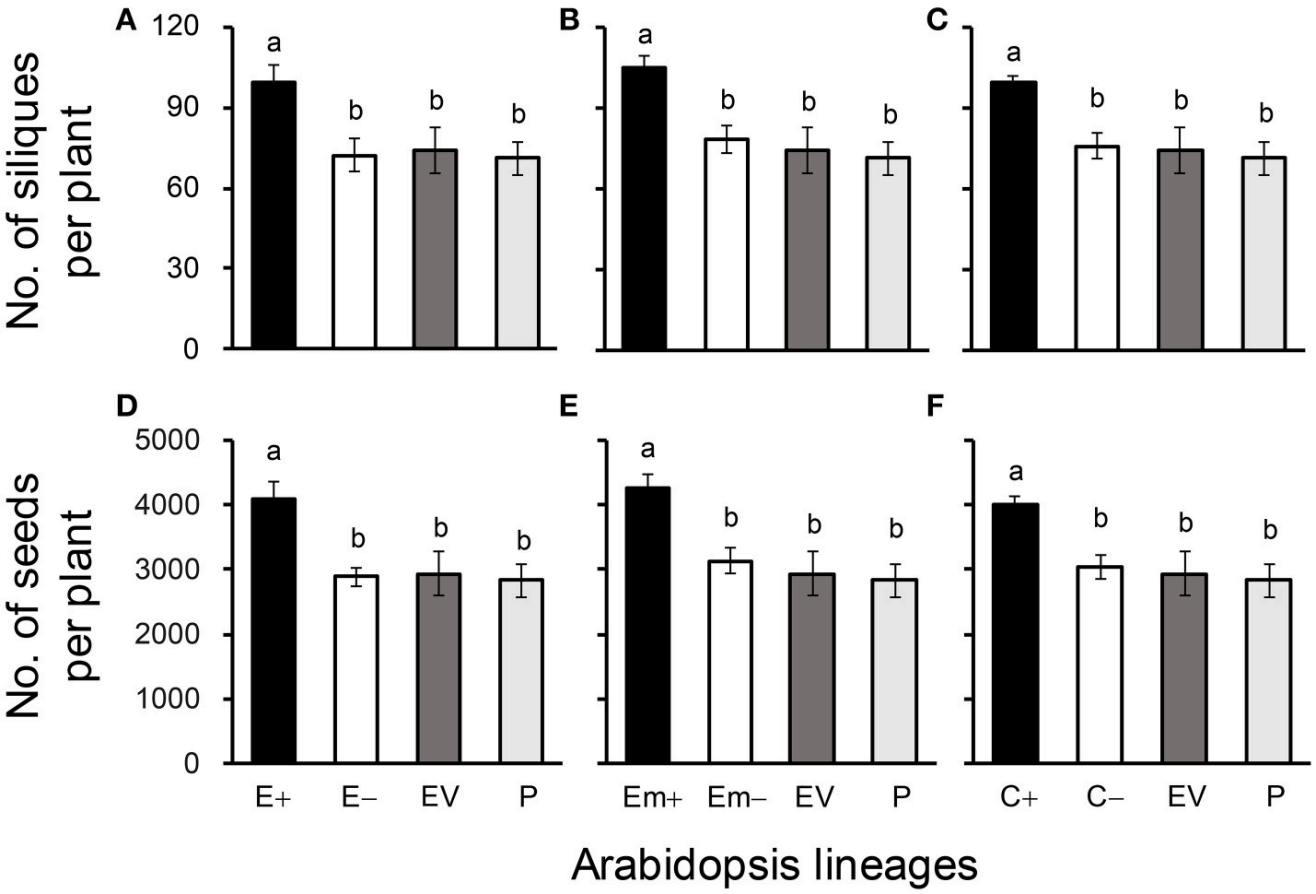
Average silique **(A–C)** and seed production **(D–F)** of three Arabidopsis transgenic events in T_3_ transgene-present, transgene-absent, empty vector (EV), and parent (P) lineages in the glyphosate-free environment. Different letters above the columns indicate significances at *P* < 0.05 based on Duncan's multiple range test (*n* = 18). E+: *EPSPS* transgene-present lineages, E–: *EPSPS* transgene-absent lineages, Em+: mutant *EPSPS* transgene-present lineages, Em–: mutant *EPSPS* transgene-absent lineages; C+: *CP4* transgene-present lineages, C–: *CP4* transgene-absent lineages. Bars represent standard errors.

**Figure 4:**
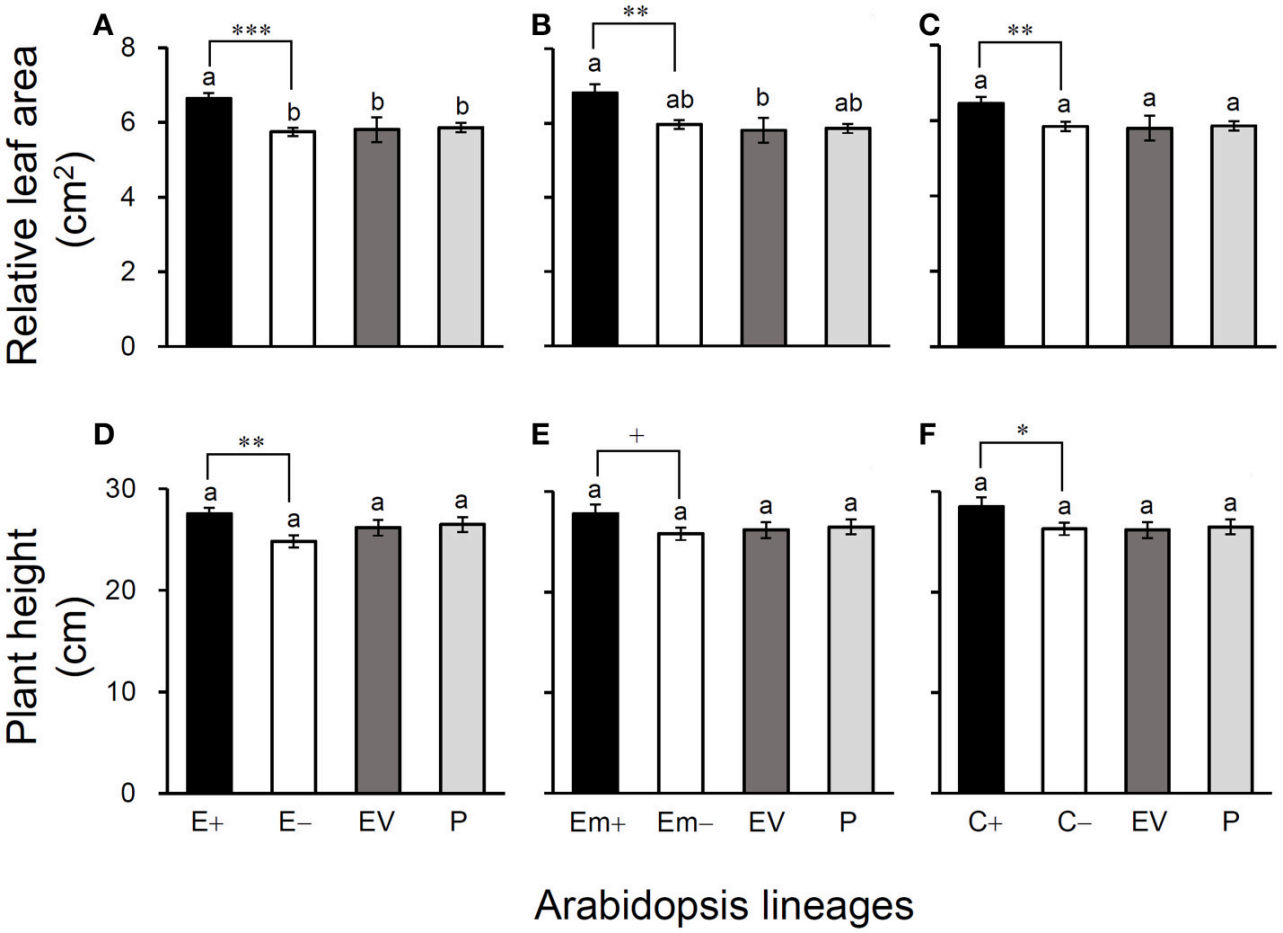
Average relative leaf-area **(A–C)** and plant height **(D–F)** of three Arabidopsis transgenic events in T_3_ transgene-present, transgene-absent, empty vector (EV), and parent (P) lineages in the glyphosate-free environment. Different letters above the columns indicate significances at *P* < 0.05 based on Duncan's multiple range test (*n* = 18). Differences between transgene-present and transgene-absent lineages were compared based on the independent *t*-test after Bonferroni correction (*n* = 18). +*P* <0.1, ^*^P < 0.05, ^**^P < 0.01, ^***^P < 0.001. E+: *EPSPS* transgene-present lineages, E–: *EPSPS* transgene-absent lineages, Em+: mutant *EPSPS* transgene-present lineages, Em–: mutant *EPSPS* transgene-absent lineages; C+: *CP4* transgene-present lineages, C–: *CP4* transgene-absent lineages. Bars represent standard errors.

All of these significant differences were in the direction of increased fitness for the transgene-present lineages relative to the controls. For example, overproduction of EPSPS was associated with increases of ~12–22% (*n* = 18) more seeds to germinate under the heat and drought stresses for the E and Em transgene-present lineages (Figures 2A–F; Table S4), although no significant differences in seed germination under the normal condition (22°C) (Table S4). Notably, the C+ transgene-present lineage showed a significant increase in seed germination under the drought stress (Figure 2F). In addition, overproduction of EPSPS resulted in 22–28% (*n* = 18) more siliques and 23–27% (*n* = 18) more seeds per plant (Figures 3A–F; Table S4). The number of branches per plant and seeds per silique was very similar across lineages (Table S4). Overall, the presence of the three transgenes was associated with larger plants (Figures 4A–F; Table S4) with enhanced fecundity (Figures 3A–F; Table S4) in the glyphosate-free environment. We did not detect evidence of fitness benefits or costs associated with the single EV lineage, compared to the parental and transgene-absent lineages.

### Biomass and auxin content

**Figure 5:**
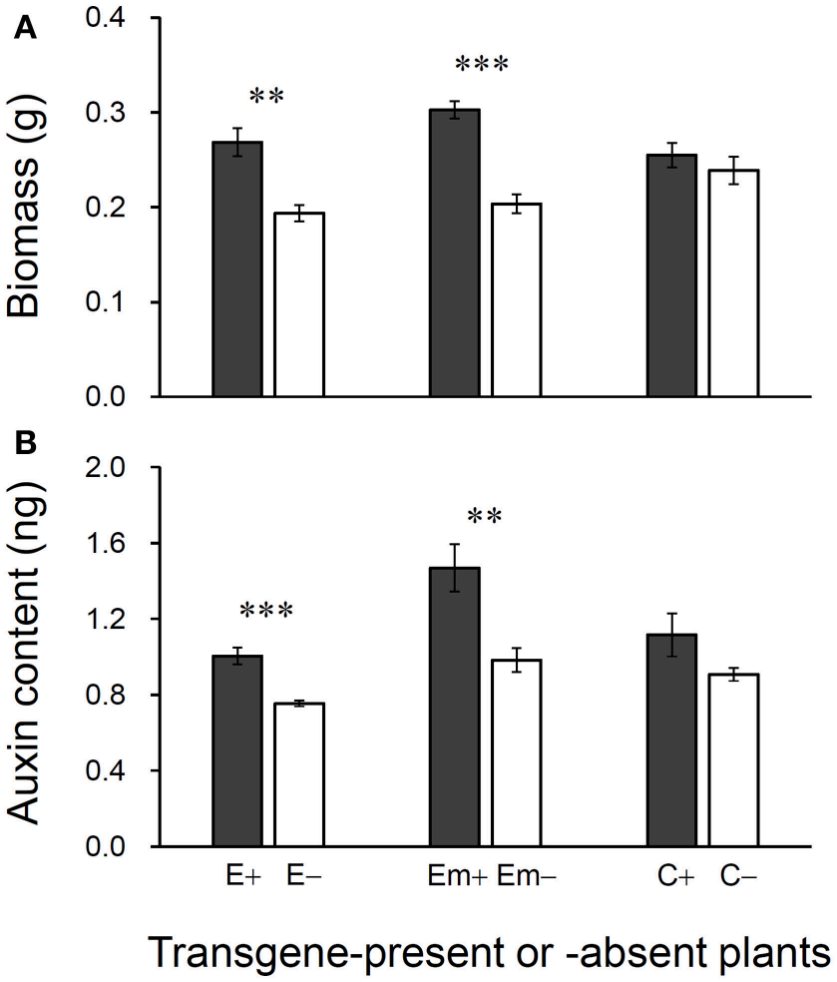
Differences in biomass **(A)** and auxin (IAA) content **(B)** of 30-day plants between T_3_ transgene-present and transgene-absent Arabidopsis lineages based on the independent *t*-test (*n* = 5). E+: *EPSPS* transgene-present lineage, E–: *EPSPS* transgene-absent lineages (event E/2); Em+: mutant *EPSPS* transgene-present lineages, Em–: mutant *EPSPS* transgene-absent lineages (event Em/3); C+: *CP4* transgene-present lineages, C–: *CP4* transgene-absent lineages (event C/2). ^**^P < 0.01, ^***^P < 0.001. Bars represent standard errors.

Significant increases in biomass and auxin content were detected in 30-day-old plants in transgene-present E+ and Em+ lineages in the T_3_ generation (Figures 5A, B). However, no significant differences in biomass or auxin content were observed between transgene-present C+ and transgene-absent C– lineages, although the values were slight higher for the C+ lineage. About 28–33% increases in biomass were observed in the transgene-present E+ and Em+ lineages, compared with their transgene-absent counterparts (Figure 5A). Increases in auxin content of around 25–33% were detected in transgene-present E+ and Em+ lineages, compared with their segregating transgene-absent counterparts (Figure 5B).

## Discussion

Results from this study demonstrated that the transfer of overexpressing *EPSPS* genes isolated from different sources, namely rice (E or Em) and *Agrobacterium* (*CP4*), into *A. thaliana* plants substantially increased their tolerance to the glyphosate (Roundup^®;^) herbicide although with considerable variation among the three *EPSPS* transgenes. This finding is based on the dose-response comparisons of >1,600 Arabidopsis plants among transgene-present lineages, their isogenic transgene-absent counterparts, the empty vector (EV), and parent controls in a climate chamber. In the dose-response experiment, all transgene-present Arabidopsis plants survived at the 0.4 m*M* glyphosate dosage, which is equivalent to the commonly used concentration of 840 g/ha for glyphosate application for agricultural weed control (Norsworthy et al., 2008). In contrast, none of the Arabidopsis plants in the corresponding transgene-absent lineages, EV, and parent controls survived at this dosage (0.4 m*M*). Increased glyphosate tolerance has also been observed in other GE plants overexpressing *EPSPS* transgenes, such as *Petunia hybrida* (Shah et al., 1986), rice (Su et al., 2008; Lu et al., 2014a,b), tobacco plants (Jones et al., 1996), and *A. thaliana* (Klee et al., 1987; Yang et al., 2017a), regardless of the origins (endogenous or exogenous) of *EPSPS* genes. All these results indicate that the transfer of an overexpressing *EPSPS* gene into plants, including the model plant Arabidopsis in this study and other studies (Klee et al., 1987; Yang et al., 2017a), can increase glyphosate tolerance of the transgenic plants due to overproduction of EPSPS.

Increased tolerance to glyphosate in our transgenic plants overexpressing an *EPSPS* gene is presumably due to the sufficiently surplus EPSPS that can bind glyphosate (Rogers et al., 1983), as reported in *P. hybrida* (Shah et al., 1986), tobacco plants (Jones et al., 1996), and Arabidopsis (Klee et al., 1987; Yang et al., 2017a). Thus, these results confirm the strategy of overproducing EPSPS driven by a strong promoter (e.g., pCaMV35S for dicots and pUbiquitin for monocots) to be effective in increase GE crops' tolerance to glyphosate, regardless of exogenous (as in this study) or endogenous (Klee et al., 1987; Su et al., 2008; Yang et al., 2017a). The application of an endogenous transgene overproducing EPSPS at a proper level to develop GE glyphosate-tolerant crops may have particular commercial values. That is, a glyphosate-tolerance transgene originating from the crop species rather than a bacterium or other sources may reduce consumers' concerns over the food safety issues (Kuiper et al., 2001; Qaim and Zilberman, 2003). In addition, the application would be particularly useful for GE crops such as sugar beets, potatoes, and vegetables, of which the consumed parts are vegetative organs, because the transgenic plants appear to have increased vigor, but with less opportunities of crop-to-wild/weed gene flow mediated by pollination.

As often happens in studies of transgenic plants, we also observed considerable variation in overexpression of the *EPSPS* genes among different transgenic events in our experiment, based on the real-time PCR analysis. Therefore, we only included transgenic events with a relatively high level of *EPSPS* expression because the main objective of this study was to determine whether overexpression of *EPSPS* genes would enhance fecundity of transgene-present plants. To address the question about the likelihood of insertion or position effect by a single transgenic event with enhanced fecundity of transgenic plants (Gressel et al., 2014; Grunewald and Bury, 2014), we included GE Arabidopsis plants of three transgenic events representing each of the three transgenic constructs with the above-average level of *EPSPS* overexpressing in our common-garden experiment for fitness comparisons. Our results did not show significant differences in fitness-related traits among the three included transgenic events of each transgene in transgene-present Arabidopsis lineages. In addition, we did not find significant differences in fitness-related traits between the EV and parental lineages. These results support previous findings that enhanced fitness/fecundity (as shown in (Su et al., 2008; Wang et al., 2014; Yang et al., 2017a),b) is not the consequence of an insertion or position effect (process of transgenesis), but due to the action of the transgene itself. We therefore confirm that overexpression of *EPSPS* in GE plants with a proper level can result in overproduction of their EPSPS and increased glyphosate tolerance.

In the common-garden experiment in a growth chamber, we observed significantly increased seed germination ratios in E+ and Em+ transgene-present lineages when seeds were exposed to the heat (28°C) and drought [200 m*M* D-mannitol (C_6_H_14_O_6_)] stresses, although no differences were found in seed germination among different lines when seeds were exposed to the normal temperature (22°C, ideal for Arabidopsis, Xiong et al., 1999). The *CP4*+ transgene-present lineages only showed significant increases in seed germination under the drought stress. Considering that auxin (IAA) can promote seed germination and plant growth under abiotic stresses (Woodward and Bartel, 2005; Liu et al., 2014; Naser and Shani, 2016), our explanation for enhanced seed germination under stresses can be attributed to the increased auxin content in transgenic Arabidopsis plants. The report of Leadem (1987) in which auxin stimulated seed germination under special conditions including heat and cold stresses supports our explanation. However, we propose more studies to test this hypothesis because very limited examples are found in the scientific literature.

In addition, our results indicated significantly greater values of a few major fitness-related traits, including the number of siliques and seeds per plant in transgene-present Arabidopsis lineages in glyphosate-free environment. Similar findings of increased fitness were also reported in transgene-present Arabidopsis plants containing a native gene overproducing EPSPS (Beres et al., in press). All these findings support our previous observation of the significantly enhanced fecundity in transgene-present crop-weed (Wang et al., 2014) and crop-wild (Yang et al., 2017b) hybrid lineages containing an *EPSPS* transgene from rice. It is apparent that the presence of a transgene overproducing EPSPS, regardless of its origin (endogenous or exogenous), may significantly enhance the fecundity of a plant. Altogether, findings from wild/weedy rice (monocot) and Arabidopsis (eudicot) indicate that overexpression of an *EPSPS* gene to a proper level with increased fecundity of GE plants in the glyphosate-free environments may be a general feature of angiosperms. Therefore, environmental impact caused by introgression of a transgene overexpressing *EPSPS* from GE glyphosate-tolerant crops into their wild/weedy relatives should be thoroughly assessed, even in the glyphosate-free environment. Further studies including hybrid descendants of transgenic crops overexpressing *EPSPS* with their wild relatives should be conducted to provide more evidence for the potential ecological impact.

What could be the underlying mechanisms for enhanced fecundity of GE plants containing an *EPSPS* overexpressing transgene in glyphosate-free environment? In this study, we detected increased auxin, an important plant growth hormone (Woodward and Bartel, 2005; Zhao, 2012; Liu et al., 2014), in transgene-present Arabidopsis lineages (E+ and Em+). We therefore hypothesize that increased total endogenous auxin may play a role in promoting the growth and development (probably also stress tolerance) of transgene-present Arabidopsis plants, eventually leading to increases in their fitness-related traits, although other factors such as enhanced photosynthetic rates by overproducing EPSPS (see Wang et al., 2014) can also promote the growth of transgene-present plants. As indicated in our previous study, significantly increased tryptophan (Trp) content was detected in four independent transgene-present crop-weed hybrid lineages overproducing EPSPS (Wang et al., 2014). Trp is an aromatic amino acid synthesized in the downstream of EPSPS in the shikimate pathway (Herrmann and Weaver, 1999; Maeda and Dudareva, 2012). Recent studies have revealed that auxin biosynthesis is a simple two-step pathway converting Trp to auxin in plants (Mashiguchi et al., 2011; Won et al., 2011). This suggests that overproduction of EPSPS may lead to increases in auxin through increased Trp (Zhao, 2012; Wang et al., 2014). Thus, the complete discovery of the precise biosynthesis pathway from EPSPS to auxin will provide deeper insight into mechanisms associated with fitness effect and environmental impact of transgenic plants that overproduce EPSPS in agricultural and natural habitats.

## Author contributions

JF: Conducted the experiment, analyzed data, and wrote the paper; PN, ZG, XG, and Y-QF: Conducted some part of the experiment; B-RL: Conceived and designed the experiment, analyzed data, and wrote the paper. All authors reviewed the manuscript.

### Conflict of interest statement

The authors declare that the research was conducted in the absence of any commercial or financial relationships that could be construed as a potential conflict of interest.
