# Supplementary material for: Overexpressing Exogenous 5-Enolpyruvylshikimate-3-Phosphate Synthase (EPSPS) Genes Increases Fecundity and Auxin Content of Transgenic Arabidopsis Plants
Source: Front Plant Sci. 2018 Feb 27;9:233. doi: 10.3389/fpls.2018.00233 (PMC5835131; doi:10.3389/fpls.2018.00233)
Supplement: Supplementary file 1 [file Image1.PDF]

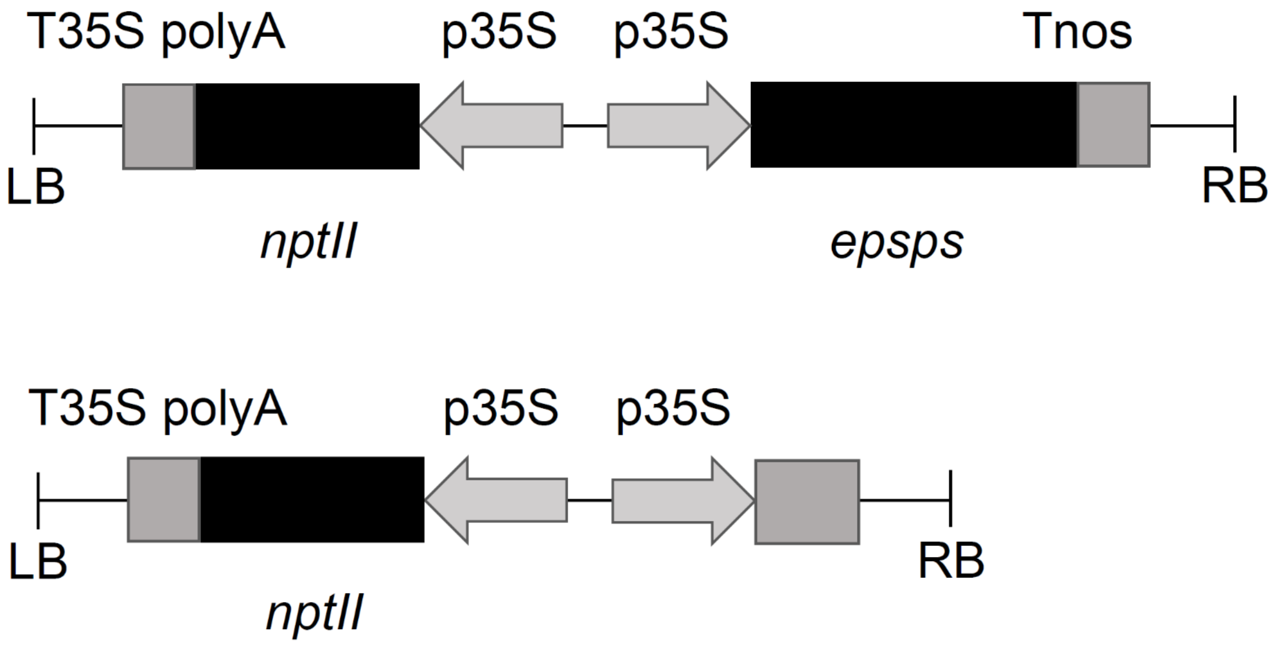

Figure S1 | Main components of the three *EPSPS* transgenic constructs (upper panel) and the empty vector (lower panel), using the pCHF3 vector for the transformation of an *Arabidopsis thaliana* strain Columbia (Col 0). LB: the left border of the constructs; 35S polyA: the terminator of the kanamycin selectable marker gene (*nptII*) driven by a cauliflower mosaic virus (CaMV) 35S promoter (p35S); the *EPSPS* transgene driven by p35S; T-nos, terminator of the *EPSPS* gene; RB: right border of the construct. The *EPSPS* transgenes are modified from the endogenous gene of cultivated rice (*Oryza sativa*) and from *Agrobacterium* sp. See Padegtte et al. (1996) [38], Su et al. (2008) [30], and Lu et al. (2014) [40] for further details of the *EPSPS* transgenes.

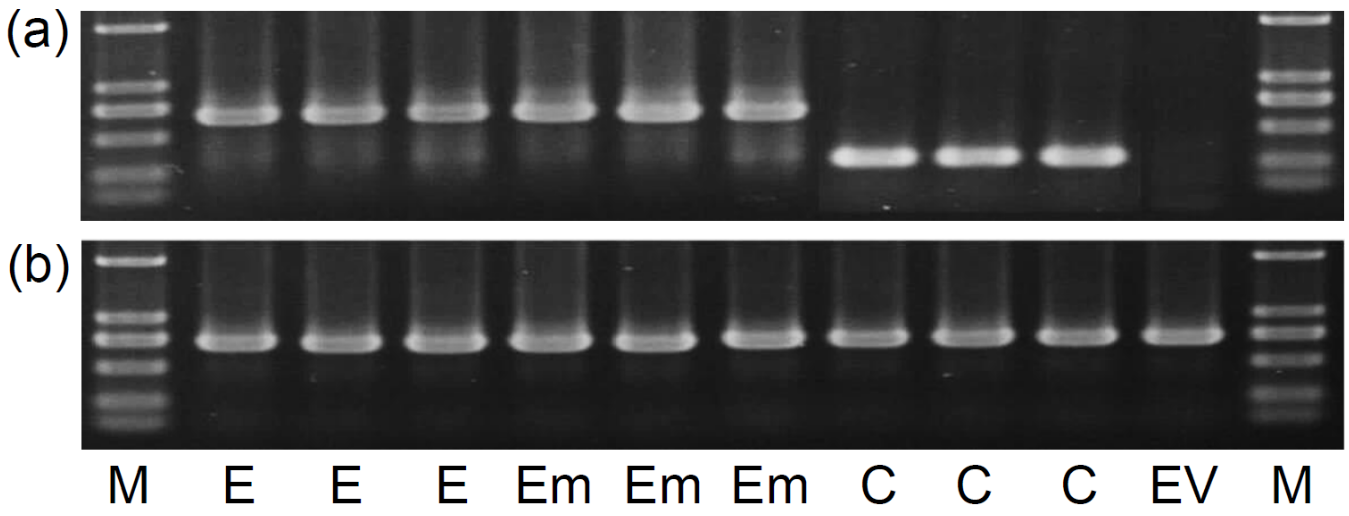

Figure S2 | Molecular identification of transgenic Arabidopsis plants based on *EPSPS* transgenes (a) and kanamycin selective marker gene (*nptII*) (b) by using polymerase chain reaction and agarose gel electrophoresis. E, *EPSPS* transgene; Em, mutant *EPSPS* transgene; C, *CP4* transgene; EV, empty vector; M, DL2000 DNA markers.

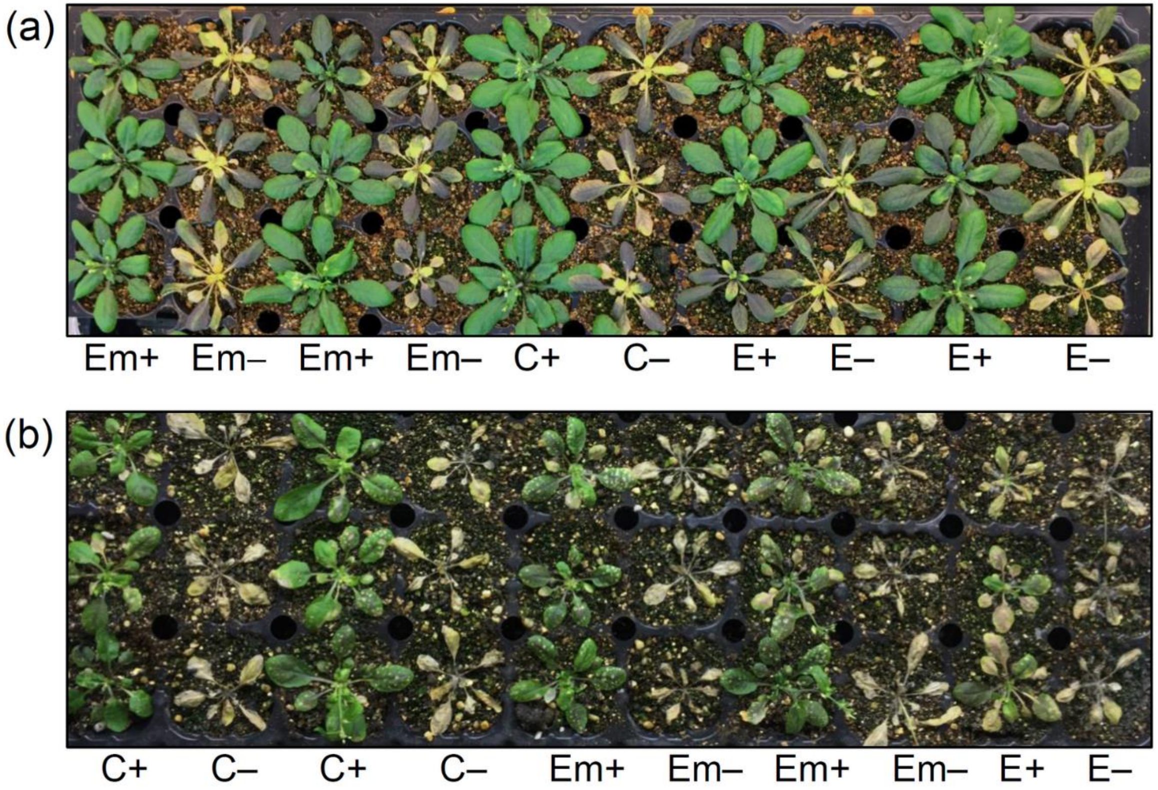

Figure S3 | Performance of the T_2 isogenic Arabidopsis seedlings with or without transgenes overexpressing EPSPS (5-enolpyruvoylshikimate-3-phosphate synthase) at the 0.4 m*M* (a) and 1.2 m*M* (b) glyphosate concentrations. E+: *EPSPS* transgene-present plants; E−: segregating transgene-absent plants; Em+: mutant *EPSPS* transgene-present plants; Em−: segregating transgene-absent plants; C+: *CP4* transgene-present plants; C−: segregating transgene-absent plants.

Table S1 | Relative expression (descent order) of *EPSPS* transgenes measured by real-time PCR (polymerase chain reaction) in different *Arabidopsis thaliana* events of the three transgenic constructs: *EPSPS* (E), mutant *EPSPS* (Em), and *CP4* (C) in T_2 and T_3 generations. Numbers in parenthesis indicate standard errors (n = 3 pooled samples, each including 8 plants). **Bold** events were selected for glyphosate resistant (T_2), and *EPSPS* expression, and fitness (T_3) experiments. **Bold** events with asterisks (*) in the T_3 generation were used to measure biomass and auxin (IAA) content.

T_2	Code of events[1]	Expression	Code of events[1]	Expression	Code of events[1]	Expression
	E+/1	7.946 (0.868)	Em+/1	7.999 (1.008)	C+/1	6.658 (0.612)
	**E+/2**	6.319 (0.521)	**Em+/2**	5.247 (0.687)	**C+/2**	5.342 (0.561)
	**E+/3**	4.271 (0.648)	**Em+/3**	4.691 (0.386)	**C+/3**	2.828 (0.237)
	**E+/4**	2.087 (0.379)	**Em+/4**	1.000 (0.210)	**C+/4**	0.817 (0.236)
	E+/5	0.750 (0.168)	Em+/5	0.634 (0.175)	C+/5	0.684 (0.210)
	E+/6	0.582 (0.120)	Em+/6	0.537 (0.153)	C+/6	0.495 (0.134)
	E+/7	0.102 (0.026)	Em+/7	0.063 (0.016)	C+/7	0.155 (0.013)
	E+/8	0.054 (0.012)	Em+/8	0.042 (0.009)	C+/8	0.138 (0.009)
	E+/9	0.030 (0.005)	Em+/9	0.011 (0.002)	C+/9	0.113 (0.010)
T_3	E+/1	7.566 (0.876)	Em+/1	8.515 (1.003)	C+/1	7.578 (0.814)
	**E+/2***	6.609 (0.943)	**Em+/2**	6.008 (0.824)	**C+/2***	6.585 (0.476)
	**E+/3**	4.354 (0.320)	**Em+/3***	4.725 (0.732)	**C+/3**	4.228 (0.226)
	**E+/4**	3.022 (0.353)	**Em+/4**	1.000 (0.479)	**C+/4**	1.331 (0.321)
	E+/5	1.817 (0.268)	Em+/6	0.784 (0.116)	C+/5	0.869 (0.163)
	E+/7	0.760 (0.162)	Em+/5	0.581 (0.058)	C+/6	0.824 (0.284)
	E+/6	0.453 (0.086)	Em+/7	0.091 (0.013)	C+/9	0.291 (0.036)
	E+/8	0.058 (0.009)	Em+/8	0.042 (0.008)	C+/7	0.204 (0.021)
	E+/9	0.031 (0.008)	Em+/9	0.017 (0.005)	C+/8	0.024 (0.006)

[1] Only transgene-present plants in the event were included for the determination of *EPSPS* gene expression.

**Table S2 | ** Method used to measure the fitness-related traits and indole-3-acetic acid (IAA) in *Arabidopsis thaliana* plants of the T_3 generation at various growth stages.

Trait	Method and time of measurement	
	Method	Time
Seed germination under normal condition (%)[1]	Ratio between the number of germinated seeds and the total number of seeds included for germination on 1/2 MS culture media at 22 °C.	Measured at the 7th day after seeds were placed on the 1/2 MS media for germination.
Seed germination under heat stress (%)	Ratio between the number of germinated seeds and the total number of seeds included for germination on 1/2 MS media at 28 °C.	Measured at the 7th day after seeds were placed on the 1/2 MS media for germination.
Seed germination under drought stress (%)	Ratio between the number of germinated seeds and the total number of seeds included for germination on 1/2 MS media with 200 mM D-mannitol ($C_6H_{14}O_6$) at 22 °C.	Measured at the 7th day after seeds were placed on the 1/2 MS media for germination.
Relative leaf area (cm^2)	Relative leaf area was determined as the average values of the length × width of the longest three leaves per plant.	Measured at the 40th day after seed germination.
Plant height at maturity (cm)	Measured from the base of the plant (above ground) to the tips of the plant.	Measured at the 90th day after seed germination.
Number of branches per plant	Total number of branches of a plant.	Measured at the 90th day after seed germination.
Number of siliques per plant	Total number of siliques of a plant.	Measured at the 90th day after seed germination.
Number of seeds per silique	Average number of seeds from 20 randomly selected siliques of a plant.	Measured at the 90th day after seed germination.
Number of seeds per plant	Total number of seeds was estimated as the number of siliques per plant × number of seeds per silique.	Measured at the 90th day after seed germination.
Seedling biomass (g)	Weight of fresh seedlings above ground from five replicates each including five plants.	Measured at the 30th day after seed germination.
Content of auxin (indole-3-acetic acid) (ng)	Average total weight of auxin in the measured plants with the same duration of growth from replicates each included a pooled sample of 5 plants.	Measured at the 30th day after seed germination.

[1]Seed germination was on 1/2 MS culture media include 30 g sucrose, 2.2 g M519 (Murashige & Skoog Basal Medium with Vitamins), and 8 g agar per liter (pH 5.7).

**Table S3 | ** One-way ANOVA to test for the effect among three transgenic events (lineages) of each transgenic construct (group, E, Em, or C) on the fitness-related traits. For seed germination, each lineage included 6 replicates (n = 6) each containing 50 seeds. For other traits, each lineage included 6 replicates (n = 6) each including 4 plants.

Trait	*EPSPS* (E)				Mutant *EPSPS* (Em)				*CP4* (C)			
	Group[1]	*df*	*F*	*P*	Group[1]	*df*	*F*	*P*	Group[1]	*df*	*F*	*P*
Seed germination under normal condition	E+	2	0.500	0.630	Em+	2	1.000	0.422	C+	2	1.000	0.422
(22 °C)	E–	2	1.000	1.000	Em–	2	3.616	0.093	C–	2	1.000	0.422
Seed germination under heat stress	E+	2	2.395	0.125	Em+	2	3.221	0.069	C+	2	11.367	0.053
(28 °C)	E–	2	2.214	0.146	Em–	2	29.509	0.051	C–	2	1.405	0.276
Seed germination under drought stress	E+	2	0.151	0.863	Em+	2	3.261	0.110	C+	2	8.704	0.077
(D-Mannitol, $C_6H_{14}O_6$, at 22 °C)	E–	2	2.957	0.128	Em–	2	32.180	0.052	C–	2	3.903	0.055
Relative leaf area	E+	2	2.245	0.140	Em+	2	0.389	0.684	C+	2	0.301	0.745
	E–	2	0.160	0.854	Em–	2	0.024	0.976	C–	2	0.961	0.405
Plant height at maturity	E+	2	0.211	0.812	Em+	2	1.201	0.328	C+	2	0.645	0.539
	E–	2	1.559	0.242	Em–	2	2.109	0.156	C–	2	2.538	0.112
Number of branches per plant	E+	2	0.373	0.695	Em+	2	0.072	0.931	C+	2	0.219	0.806
	E–	2	0.372	0.695	Em–	2	2.390	0.126	C–	2	2.705	0.104
Number of siliques per plant	E+	2	1.057	0.372	Em+	2	0.345	0.714	C+	2	0.184	0.834
	E–	2	3.116	0.074	Em–	2	2.532	0.113	C–	2	1.953	0.176
Number of seeds per silique	E+	2	0.264	0.772	Em+	2	1.024	0.383	C+	2	0.449	0.647
	E–	2	0.193	0.827	Em–	2	0.047	0.954	C–	2	1.801	0.199
Number of seeds per plant	E+	2	1.277	0.307	Em+	2	0.790	0.472	C+	2	0.235	0.793
	E–	2	3.058	0.079	Em–	2	2.646	0.104	C–	2	1.777	0.208

[1] +: transgene-present groups, –: transgene-absent groups.

Table S4 | Comparison of differences in fitness-related traits between Arabidopsis transgene-present (+) and transgene-absent (–) groups, each containing three transgenic events (lineages) with 18 replicates (n = 18), based on the *t*-test with Bonferroni corrections. For seed germination, each replicate had 50 seeds; for other traits, each replicate had 4 plants. Numbers in parenthesis indicate standard errors. + P <0.1; * P <0.05; ** P <0.01; *** P <0.001.

Trait	Group[1]	*EPSPS* (E)	*Mutant EPSPS* (Em)	*CP4* (C)
Seed germination under normal condition (%)	+	99.77 (0.11)	99.89 (0.11)	99.89 (0.11)
	–	100 (0)	99.32 (0.68)	99.89 (0.11)
Seed germination under heat stress (%)	+	98.64 (0.62) ***	98.63 (0.97) ***	87.98 (3.03)
	–	86.42 (3.31)	81.54 (7.36)	83.23 (8.80)
Seed germination under drought stress (%)	+	99.41 (0.13) ***	96.23 (2.09) **	86.21 (5.11)
	–	86.50 (3.37)	74.76 (3.31)	74.87 (1.85)
Relative leaf area (cm^2)	+	6.63 (0.15) ***	6.81 (0.24) **	6.46 (0.16) **
	–	5.75 (0.11)	6.07 (0.12)	5.84 (0.13)
Plant height at maturity (cm)	+	27.47 (0.62) **	27.79 (0.88) +	28.49 (0.53) *
	–	24.82 (0.59)	25.80 (0.63)	26.31 (0.66)
Number of branches per plant	+	7.76 (0.20)	8.14 (0.28)	8.00 (0.18)
	–	7.35 (0.21)	7.71 (0.21)	7.67 (0.28)
Number of siliques per plant	+	99.39 (6.36) **	104.88 (4.33) **	99.04 (2.37) **
	–	72.08 (3.93)	78.27 (4.99)	75.72 (4.50)
Number of seeds per silique	+	41.04 (0.37)	40.76 (0.29) *	40.36 (0.30)
	–	40.19 (0.42)	40.01 (0.17)	40.14 (0.15)
Number of seeds per plant	+	4082.86 (265.74) ***	4271.74 (209.97) **	4013.64 (111.45) *
	–	2882.96 (145.04)	3130.07 (199.79)	3110.38 (261.95)

[1] +: transgene-present groups, –: transgene-absent groups.
